# Enhanced TiO_2_-Based Photocatalytic Volatile Organic Compound Decomposition Combined with Ultrasonic Atomization in the Co-Presence of Carbon Black and Heavy Metal Nanoparticles

**DOI:** 10.3390/molecules29163819

**Published:** 2024-08-12

**Authors:** Zen Maeno, Mika Nishitani, Takehiro Saito, Kazuhiko Sekiguchi, Naoki Kagi, Norikazu Namiki

**Affiliations:** 1 School of Advanced Engineering, Kogakuin University, 2665-1, Nakano-machi, Hachioji 192-0015, Japan; 2 Graduate School of Science and Engineering, Saitama University, 255 Shimo-Okubo, Sakura 338-8570, Japan; 3 School of Environment and Society, Tokyo Institute of Technology, 12-1, Ookayama-2, Meguro-ku, Tokyo 152-8552, Japan

**Keywords:** volatile organic compound, photocatalytic degradation, carbon black, heavy metal nanoparticles, ultrasonic atomization

## Abstract

Volatile organic compounds (VOCs) are representative indoor air pollutants that negatively affect the human body owing to their toxicity. One of the most promising methods for VOC removal is photocatalytic degradation using TiO_2_. In this study, the addition of carbon black (CB) and heavy metal nanoparticles (NPs) was investigated to improve the efficiency of a TiO_2_-based photocatalytic VOC decomposition system combined with ultrasonic atomization and ultraviolet irradiation, as described previously. The addition of CB and Ag NPs significantly improved the degradation efficiency. A comparison with other heavy metal nanoparticles and their respective roles are discussed.

## 1. Introduction

Volatile organic compounds (VOCs) are representative indoor air pollutants that negatively affect the human body owing to their toxicity and carcinogenicity. These pollutants are closely related to the sick building syndrome, including mucous membrane irritation, headaches, and fatigue [1,2]. Photocatalytic degradation is one of the most promising methods for VOC removal [3,4,5,6]. TiO_2_-photocatalyzed systems have been extensively studied because of their potential for operation under ultraviolet (UV, typically UV-A) irradiation without external heating. The presence of water molecules in the gas phase is effective in maintaining catalytic activity, and the formation of OH radicals is the key to achieving high degradation efficiency. However, hydrophobic VOCs such as toluene are less reactive than hydrophilic VOCs in TiO_2_-photocatalyzed systems. A combined ozone oxidation and photocatalytic degradation system has also been studied [7,8,9,10,11]. Despite the effective degradation of gaseous organic compounds, these systems still suffer from several problems related to their strong dependence on relative humidity [12], the interference of intermediate degradation products with the degradation process [13,14], and catalyst deterioration owing to the adsorption of intermediates [13,15]. In addition, the handling of ozone has some complexity due to its toxicity under high concentration conditions. Although a combined system using shorter wavelength UV-C has also been studied to generate OH radical effectively [16,17,18], the in situ formation of ozone is concerned. Therefore, developing other methods to effectively accelerate photocatalytic reactions is still desirable.

Sekiguchi et al. proposed a method for the decomposition of VOCs using mists containing TiO_2_ particles generated by the ultrasonic (US) irradiation of a TiO_2_ particle suspension [19,20]. Ultrasonically generated mists range from nanometer to micrometer and allow the transportation of solid materials in the gas phase depending on the size of the mists [21,22]. This method is applicable to the continuous decomposition of toluene as a model hydrophobic VOC by increasing the gas–liquid interphase owing to atomization. A possible application of this system is a humidifying air purifier with degradation functionality. In this system, ultrasonically generated mists react with gas-phase toluene to produce water-soluble intermediates that are dissolved in the TiO_2_ suspension phase and then further decomposed. The incorporation of the water-soluble intermediate products into the droplet is the key to maintaining a stable photocatalytic decomposition rate.

In this study, we aimed to improve the degradation efficiency of the aforementioned systems by combining them with additives such as carbon black (CB) and heavy metal nanoparticles (NPs). Carbonaceous materials are well-known adsorbents for capturing hydrophobic organic molecules, possibly incorporating the intermediate product in a water phase [3], and heavy metal NPs have the potential to improve the photocatalytic efficiency of TiO_2_ systems [23,24]. The addition of both CB and Ag NPs was the best combination for improving the degradation efficiency in the gas and water phases. The roles of CB and Ag NPs are discussed.

## 2. Results and Discussion

**Figure 1:**
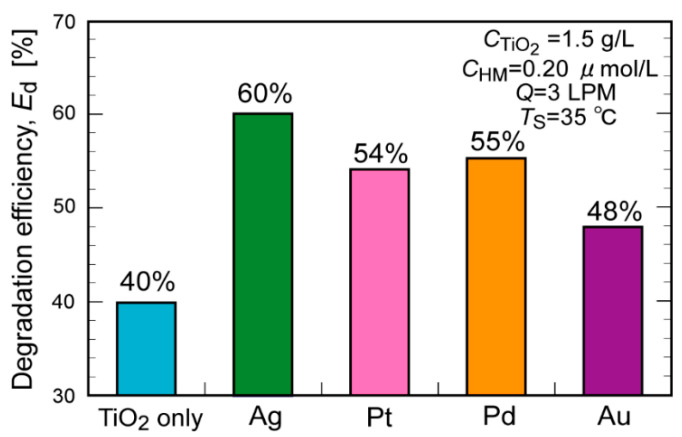
Comparison of the toluene degradation efficiencies with and without the addition of different types of heavy metal nanoparticles to the TiO_2_ suspension.

**Figure 2:**
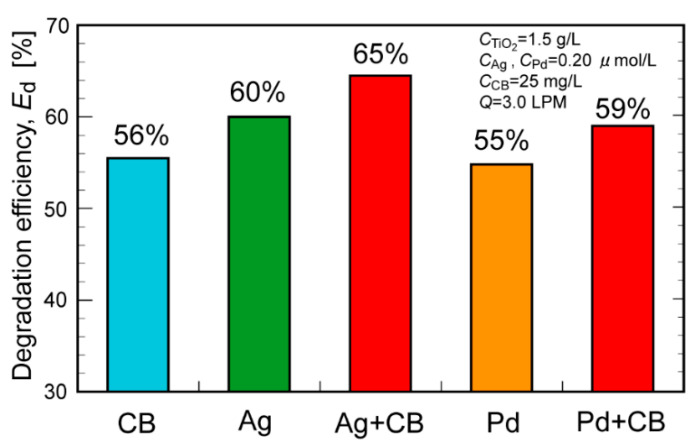
Comparison of the toluene degradation efficiencies with and without addition of CB and/or heavy metal (Ag or Pd) nanoparticles to the TiO_2_ suspension.

Figure 1 shows the toluene degradation efficiency, *E*_d_, obtained in the absence (denoted as TiO_2_ only) and presence of heavy metal NPs (denoted as Ag, Pt, Pd, and Au). The addition of a very small amount of metal NPs enhanced the *E*_d_ from 40% to 48–60%. The *E*_d_ values increased in the following order: Ag > Pd > Pt > Au > TiO_2_ only. Next, CB was added to the TiO_2_ suspension (denoted as CB) or the Ag- or Pd NP-containing suspension (denoted as Ag+CB and Pd+CB, respectively). In all cases, the addition of CB increased the *E*_d_ by approximately 5% (from 40%, 60%, and 55% to 56%, 65%, and 59%, respectively; Figure 2). The addition of both heavy metal NPs and CB effectively improved the *E*_d_. The lower effectiveness of metal NPs in the co-presence of CB compared (approximately 5% increase) might be due to the lower toluene concentration at the high-level conversion region and resulting lower mass transfer. The XRD measurements of TiO_2_ (as received) before and after the addition of Ag NPs and under UV irradiation or not were performed. In all cases, only the XRD pattern derived from anatase and rutile was observed (Figure S1).

**Figure 3:**
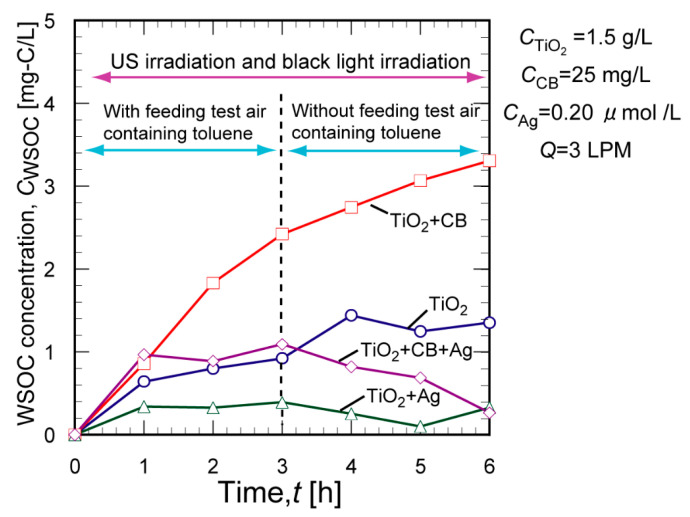
Change in the WSOC concentration in the TiO_2_ suspension with and without additives (CB or Ag).

To gain insight into the degradation reaction and the effects of CB and Ag NPs, the formation of water-soluble organic compounds (WSOCs) was monitored under four sets of reaction conditions: TiO_2_ only, TiO_2_+CB, TiO_2_+Ag, and TiO_2_+CB+Ag. Toluene vapor was fed from 0 to 3 h while monitoring the concentration of the WSOCs, *C*_WSOC_. After 3 h of reaction, toluene feeding was stopped, while the *C*_WSOC_ was continuously monitored. Figure 3 shows the time course of the *C*_WSOC_ in the TiO_2_ suspension with and without additives (CB or Ag). Without any additives (TiO_2_ only), after 3 h, the C_WSOC_ was approximately 0.9 mg-C/L and then slightly increased to approximately 1.0 mg-C/L after stopping toluene vapor feeding. In the presence of CB (TiO_2_+CB), after 3 h, the *C*_WSOC_ was approximately twice as high as that without any additives (approximately 1.8 mg-C/L), and the *C*_WSOC_ monotonically increased from approximately 1.8 to 2.4 mg-C/L despite the absence of toluene vapor feeding. Toluene was adsorbed on the CB during vapor feeding and then decomposed into water-soluble intermediates, increasing the *C*_WSOC_ after stopping toluene feeding. When Ag NPs were added to the above two reaction systems (TiO_2_+Ag and TiO_2_+CB+Ag), the *C*_WSOC_ decreased after stopping toluene vapor feeding, resulting in much lower *C*_WSOC_ values (below 0.5 mg-C/L) compared to the cases in the absence of Ag (TiO_2_ only and TiO_2_+CB). These results clearly show that Ag NPs accelerate the TiO_2_ photocatalytic degradation of toluene, especially the successive decomposition of water-soluble intermediates into mineralized products.

**Figure 4:**
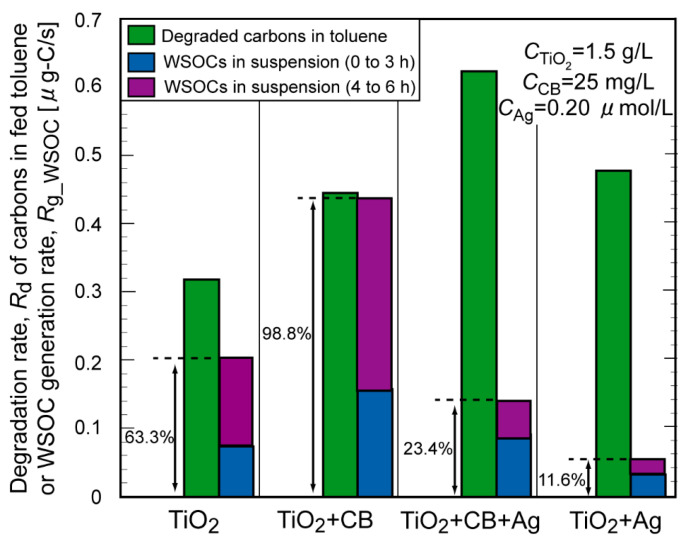
Comparison of the decomposed carbon amounts in fed toluene and generated WSOC in the TiO_2_ suspension with and without additives.

We also calculated the toluene decomposition rate normalized by carbon numbers (*R*_d_) during toluene vapor feeding (0–3 h), as well as the formation rate of WSOC (*R*_g___WSOC_) under toluene feeding (0–3 h) and after stopping the feeding (3–6 h). The *R*_d_ and *R*_g___WSOC_ were compared for four reaction conditions: TiO_2_ only, TiO_2_+CB, TiO_2_+CB+Ag, and TiO_2_+Ag (Figure 4). The addition of CB increased the *R*_g___WSOC_. The ratio of the summed *R*_g___WSOC_ (determined by the summation of the values at 0–3 h and 3–6 h) to the *R*_d_ was also calculated (denoted as *f*_WSOC_). The *f*_WSOC_ value for TiO_2_+CB was calculated as 98.8%, which is much higher than that for TiO_2_ alone (63.3%), confirming that the TiO_2_ suspension promotes the degradation of water-soluble intermediates, whereas the main role of CB is likely the adsorption of toluene rather than the promotion of decomposition reactions. The addition of Ag NPs to TiO_2_ and TiO_2_+CB systems decreased the *f*_WSOC_ from 63.3% to 11.6% and from 98.8% to 23.4%, respectively, which supports the view that Ag NPs enhanced the degradation of water-soluble intermediates to mineralized products. In conclusion, the enhanced degradation efficiency of the TiO_2_+CB+Ag system was ascribed to the combination of toluene adsorption by CB and the acceleration of the TiO_2_-photocatalyzed degradation of water-soluble intermediates.

**Figure 5:**
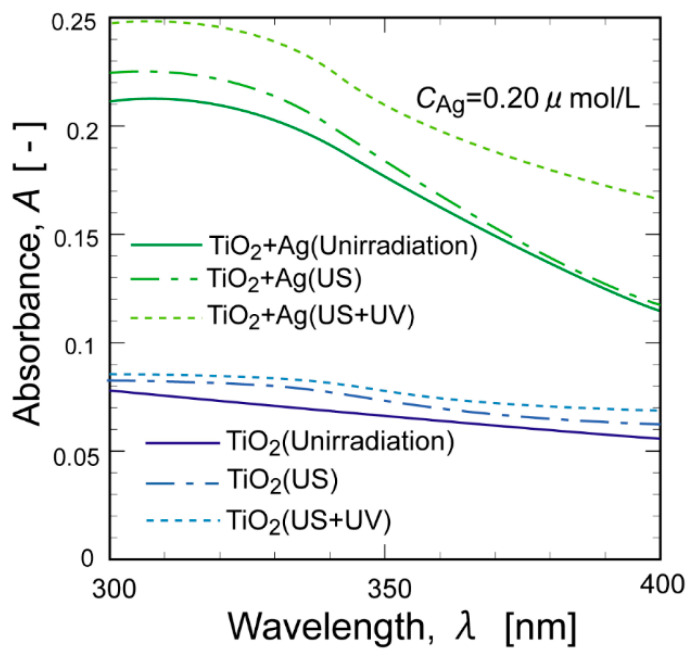
UV-vis spectra of the TiO_2_ suspension in the presence or absence of Ag NPs under irradiation with US and/or UV.

To investigate the adsorption properties of the series of TiO_2_ suspensions, UV-vis measurements were performed. Figure 5 shows the UV-vis spectra for a series of TiO_2_ suspensions, including one containing Ag NPs, after US and UV irradiation. Regardless of the presence or absence of Ag NPs, US irradiation induced an increase in absorbance in the UV-vis spectra owing to the enhancement of dispersiveness. UV irradiation further increased the absorbance, especially for the TiO_2_ suspension containing Ag NPs (TiO_2_+Ag), indicating that the oxidized surface of the Ag NPs was photoreduced by UV irradiation [25]. The UV-vis spectra of the TiO_2_ suspensions are shown in Figure S2. The addition of heavy metal NPs resulted in enhanced absorbance in the UV-vis spectra. The absorbance at 360 nm increased in the following order: Ag > Pd > Au > Pt > TiO_2_. This order is slightly different from that of the *E*_d_ (Ag > Pd > Pt > Au > TiO_2_).

**Figure 6:**
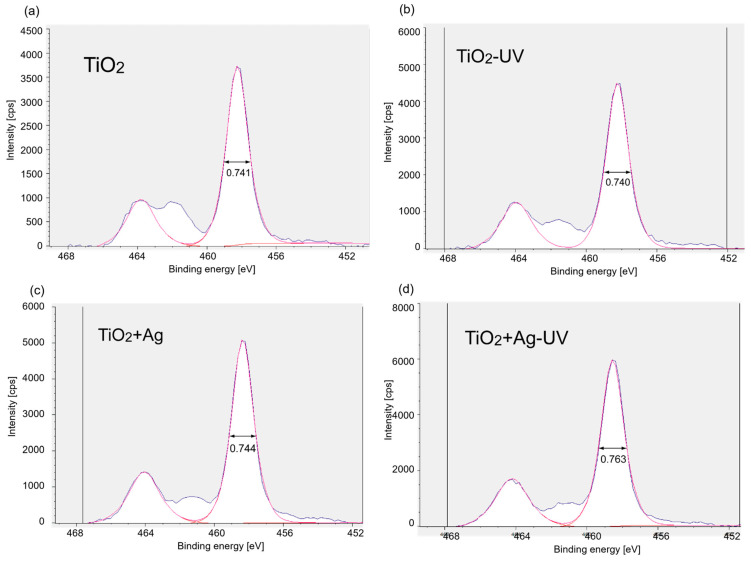
XPS spectrum of the Ti 2p peak for TiO_2_ suspension samples with and without Ag particles before and after UV irradiation Blue and red line shows raw and fitted date. (**a**) TiO_2_, (**b**) TiO_2_ after UV irradiation, (**c**) TiO_2_+Ag, and (**d**) TiO_2_+Ag after UV irradiation.

**Figure 7:**
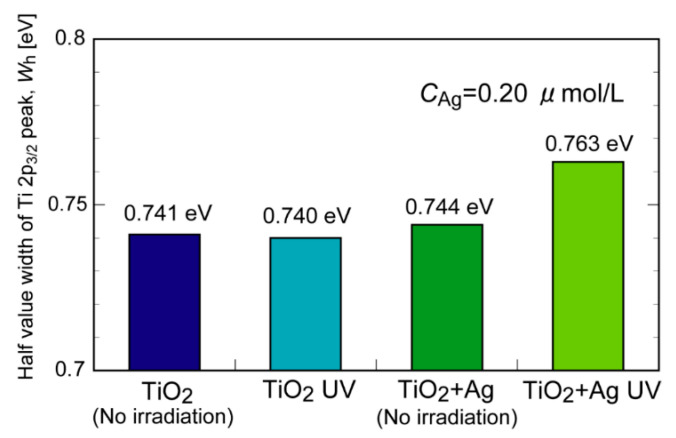
Comparison of the full width at half maximum (WFHM) values of the Ti 2p_3/2_ peak for TiO_2_ suspension samples with and without Ag particles before after UV irradiation.

We also performed X-ray photoelectron spectroscopy (XPS) measurements to study the surface chemical states of TiO_2_ before and after UV irradiation. Figure 6a–d show the XPS spectra for the Ti 2p region for a series of TiO_2_ samples. The spectra for the O 1s are also shown in Figure S3. All the XPS spectra exhibited peaks at approximately 464 and 458 eV assignable to the Ti 2p_1/2_ and 2p_3/2_ states, respectively, which are derived from the Ti^4+^ species on TiO_2_. For TiO_2_ without metal NPs, the peak position and full width at half maximum (WFHM) values for Ti 2p_2/3_ were nearly the same, regardless of the presence or absence of UV irradiation (0.741 and 0.740 eV for TiO_2_ and TiO_2_-UV, respectively, Figure 7). In the case of the Ag NP-containing TiO_2_, the WFHM value increased from 0.744 to 0.763 eV upon UV irradiation, indicating that the Ti 2p_3/2_ peak broadened. Ag NPs capture electrons generated from TiO_2_ by UV irradiation and inhibit the recombination of electrons and holes [26]. In addition, previous reports have found that the Ti 2p_3/2_ peaks were broadened in the XPS spectra of Ag-loaded TiO_2_ prepared by photoreduction because some of the surface Ti^4+^ species were reduced to Ti^3+^ by trapped electrons [25]. In our case, the added Ag NPs were immobilized on the TiO_2_ surface, promoting the reduction in the surface Ti^4+^ species in a similar manner.

**Figure 8:**
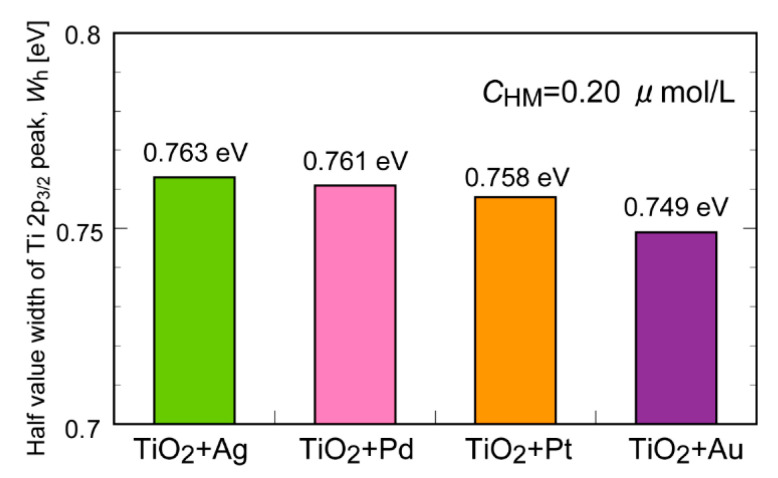
Comparison of the WFHM values of the Ti 2p_3/2_ peak for samples of TiO_2_ suspension with the addition of different types of heavy metal nanoparticles after UV irradiation.

**Figure 9:**
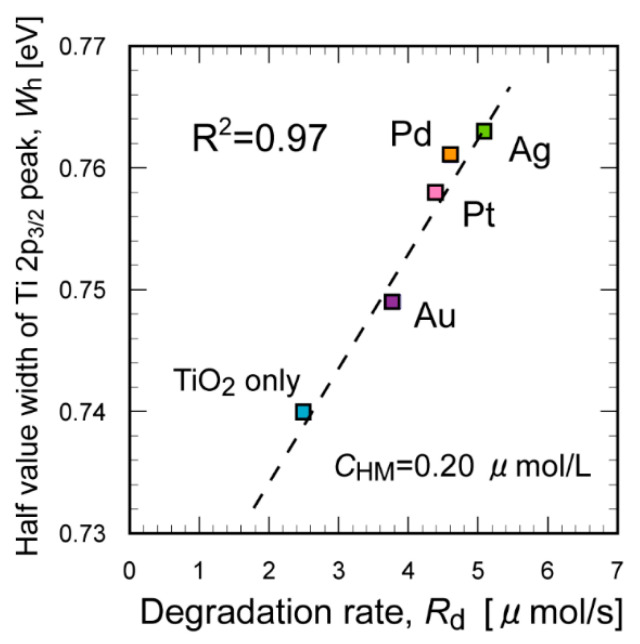
Correlation between the degradation rate, *R*_d_, and the WFHM values of the Ti 2p_3/2_ peak for TiO_2_ suspension samples with the addition of different heavy metal NPs after UV irradiation.

We also performed a similar XPS analysis for other metal (Pd, Au, and Pt) NP-containing TiO_2_. A similar increase in the WFHM value was observed after UV irradiation, although the degree of increase was lower than that for Ag NPs (Figure S4). The WFHM values increased in the same order as the *E*_d_ (i.e., Ag > Pd > Pt > Au, Figure 8). This result differs from the discussion of the UV-vis measurements (see above). To further investigate the correlation between the photocatalytic degradation performance and the surface chemical state, the WFHM value was plotted as a function of the toluene degradation rate, *R*_d_. Good correlation was found between the WFHM value and *R*_d_ (Figure 9, R^2^ = 0.97), indicating that capturing the electrons generated from TiO_2_ by UV irradiation is the key to enhancing the degradation efficiency. Holes in TiO_2_ generated by UV irradiation are known to react with H_2_O to give OH radicals, which further react with organic molecules to promote degradation reactions [27]. The added Ag NPs effectively inhibited electron–hole recombination, serving as the most effective heavy metal NPs (Figure S5). The generated OH radicals effectively decompose toluene and WSOC intermediates captured by CB, resulting in the enhanced degradation efficiency.

## 3. Materials and Methods

### 3.1. Photocatalytic Degradation of Toluene

**Figure 10:**
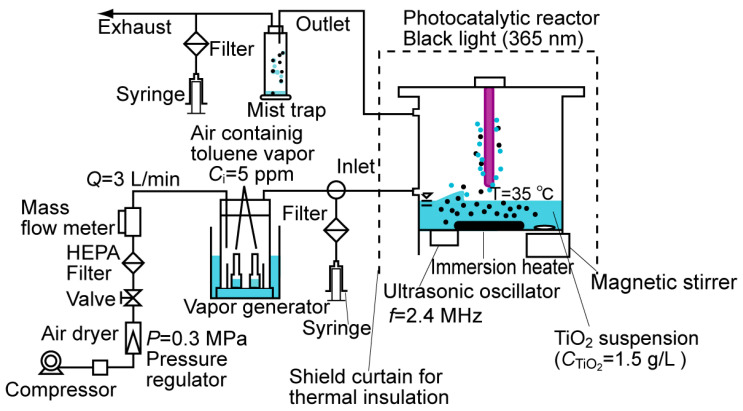
Setup for the photocatalytic decomposition of toluene vapor by TiO_2_-containing droplets generated by the ultrasonic atomization technique.

The experimental setup for the degradation of toluene (as a model VOC) using ultrasonically generated mists containing TiO_2_ is shown in (Figure 10). The experimental reactor, which consisted of poly(methyl methacrylate) resin (Figure S6), was equipped with an ultrasonic transducer (Honda Electronics, Toyohashi, Japan, HM-303N). Ultrasonically generated mists containing TiO_2_ were generated inside the reactor when a TiO_2_ suspension in deionized water was irradiated with 2.4 MHz ultrasound waves. Degussa P-25 TiO_2_ (Nippon Aerosil, Tokyo, Japan) was used in all the experiments because the P-25 TiO_2_ particles can generate a sufficient amount of OH radicals in the liquid phase [28]. The crystal structure of the P-25 TiO_2_ particles was approximately 80% anatase and 20% rutile, and the average particle diameter was approximately 30 nm. The submicrometer-sized aggregation of TiO_2_ was observed by scanning electron microscopy (SEM) (Figure S7). The surface area of TiO_2_, as measured with a BET surface analyzer (Micromeritics, Norcross, GA, USA, Flowsorb III-2305), was 50 m^2^g^−1^. The TiO_2_ concentration was fixed at 1.5 g L^−1^. A black light blue (BLB) lamp with a maximum light intensity output of 365 nm (UV_365_; Sankyo Denki, Hiratsuka, Japan, FL4BLB) was used as the light source. Detailed emission spectra of the lamps are presented elsewhere [19]. The observed relative humidity was over 95%, which was outside of the measurement range for all conditions, indicating that the mist in the reactor could be maintained under stable conditions.

Dry air containing 5 ppm of toluene vapor was introduced into the reactor at 3.0 L min^−1^ from the bottom side of the reactor. Before the reaction, TiO_2_ suspension was irradiated by a US wave under black light irradiation at 35 °C to remove the pollutants on TiO_2_ surfaces and/or in aqueous phase. Toluene vapor was then introduced for 30 min to obtain a stable toluene concentration. After stabilizing the toluene concentration at 5 ppm, the degradation reaction was started (*t* = 0 min).

In this study, CB (Mitsubishi Chemical Corporation, Tokyo, Japan, MA100) and/or heavy metal NPs, such as Ag (particle size: 5–30 nm), Pt (1–6 nm), Au (1–4 nm), and Pd (2–7 nm) (Renaissance Energy Research Co., Ltd., Osaka, Japan, as a dispersed solution), were introduced into the reaction system. The experimental schedule is shown in Figure S8. To compare the *E*_d_ after the addition of the additives with that before the addition of the additives, an experiment was first conducted using only a suspension of TiO_2_ particles, and then a similar experiment was repeated with the addition of the additives. The amounts of CB and metal NPs were 25 mg/L and 20 μmol/L, respectively. The *E*_d_ was determined as follows:

(1) $$E_{d}\left[\%\right]=(1-\frac{C_{out}\left[ppm\right]}{C_{in}\left[ppm\right]})\times 100$$

### 3.2. Determination of the Amount of WSOCs

Ultrapure water treated by UV irradiation (ADVANTEC, Tokyo, Japan, RFU464CC) was used as the suspension to reduce the TOC concentration (approximately 5 ppb). The suspension (5 mL) in the reaction tank was collected from the sampling tube using a syringe (TERUMO, Tokyo, Japan, SS-10SZP) through a cartridge filter (ADVANTEC; 25HP045AN). The reaction conditions were the same as those for the photocatalytic degradation experiments.

The suspension for WSOC concentration measurement was collected using a syringe through a cartridge filter every 1 h during UV irradiation for 6 h (*t* = 0–6 h). The air supply containing toluene was stopped at *t* = 3 h, whereas samples were collected every 1 h in the same manner until *t* = 6 h. As a pretreatment procedure, 5 mL of each water sample in the suspension was diluted to 50 mL with ultrapure water and analyzed using a TOC analyzer. When the experiments were conducted in the presence of additives, the experiments were first conducted with a TiO_2_ particle suspension only. After confirming the stabilization of degradation efficiency, additives were added, and the experiments and sample collection were performed in the same manner. The schedule of these experiments is shown in Figure S9.

For comparison of the amount of carbon in the decomposed toluene with the amount of WSOCs present in the suspension as determined by a TOC meter, the *R*_d_ [μg-C/s] and *R*_g_WSOC_ [μg-C/s] were calculated using the following equations.

(2) $$R_{d}\left[\mu g-C/s\right]=E_{d}\times C_{in}\left[g/m^{3}\right]\times Q\left[m^{3}/s\right]\times \left(\frac{M_{C7}\;[g-C/mol]}{M_{toluene}\;[g/mol]}\right)\times 10^{6}$$

(3) $$R_{g\_WSOC}\left[\mu g-C/s\right]=\left(\frac{{\bar{C}}_{WSOC}\left[g/L\right]\times V[L]}{t\;[s]}\right)\times 10^{6}$$

where *M*_C7_, *M*_toluene_, ${\bar{C}}_{WSOC}$, and *V* denote the molar mass of seven carbons, molar mass of toluene, average WSOC concentration in the suspension at *t* = 0–3 h, and suspension volume, respectively. The *f*_WSOC_ was then calculated from the *R*_d_ and *R*_g_WSOC_ as follows:

(4) $$f_{WSOC}[\%]=\frac{R_{g\_WSOC}\;[\mu g-C/s]}{R_{d}\;[\mu g-C/s]}\times 100$$

### 3.3. XPS Analysis of the TiO_2_ Suspension

Approximately 70 mL of the TiO_2_ suspension was transferred from the photocatalytic reactor to a filter (ADVANTECH, GC-50) to separate the water by vacuum filtration. The obtained solid sample was dried in a desiccator for at least 24 h and used for XPS analysis. Heavy metal NP-containing TiO_2_ samples were prepared in the same manner. For the UV-irradiated samples, the TiO_2_ suspension was irradiated with BLB lamp in the reactor, and UV irradiation was performed immediately before the XPS sample preparation (Figure S10). XPS spectra were measured on a JEOL (Tokyo, Japan) JPS-9030 spectrometer having a modified UHV chamber employing Mg Kα radiation. Charge correction was performed using the O 1s peak at 532.0 eV.

## 4. Conclusions

In conclusion, CB and Ag NPs are effective additives for the TiO_2_-photocatalytic decomposition of toluene under ultrasonic atomization. The *R*_d_ was enhanced by a factor of approximately two in the co-presence of CB and Ag NPs. A detailed analysis of the WSOC concentration indicates that the roles of CB and Ag NPs in improving the degradation efficiency are different. CB effectively accumulated WSOC intermediates in the aqueous phase, whereas Ag NPs accelerated the decomposition of WSOCs. The XPS analysis of TiO_2_ after the addition of different heavy metal NPs followed by UV irradiation revealed a reduction in the surface species, possibly owing to the electrons generated from TiO_2_ by UV irradiation, as indicated by the increase in the WHFM of the Tip_2/3_ peak. This value increased in the same order as the *E*_d_ as follows: Ag > Pd > Pt > Au > TiO_2_ (without NPs), implying that Ag NPs effectively capture the electrons derived from the UV irradiation of TiO_2_, thus suppressing the electron–hole recombination and improving the photocatalytic degradation efficiency.

## Data Availability

The data presented in this study are available on request from the corresponding author.

## Supplementary Materials

The following supporting information can be downloaded at: https://www.mdpi.com/article/10.3390/molecules29163819/s1, Figure S1: XRD patterns of TiO_2_ (as received), the one after addition of Ag NPs and/or UV irradiation; Figure S2: UV-vis spectra of TiO_2_ suspension containing heavy metal NPs after US and UV irradiation; Figure S3: XPS spectra of O 1s of a series of TiO_2_ sample; Figure S4: XPS spectrum of Ti 2p peak for samples of TiO_2_ suspension containing Pd, Pt, or Au NPs after UV irradiation; Figure S5: A possible role of Ag NPs in enhancement of toluene degradation reaction in US-generated suspension containing TiO_2_ under UV irradiation; Figure S6: Dimensional diagram of the photocatalytic reactor; Figure S7: SEM image of TiO_2_ (as-received); Figure S8: Time schedule of each run of the photocatalytic degradation experiments with feeding of additives into TiO_2_ suspension; Figure S9: Time schedule of each run of experiments on sampling for WSOC concentration determination (a) without or (b) with additive; Figure S10: Procedures to prepare samples for analyses by XPS analyzer.
