# Supplementary material for: Enhanced TiO2-Based Photocatalytic Volatile Organic Compound Decomposition Combined with Ultrasonic Atomization in the Co-Presence of Carbon Black and Heavy Metal Nanoparticles
Source: Molecules. 2024 Aug 12;29(16):3819. doi: 10.3390/molecules29163819 (PMC11357467; doi:10.3390/molecules29163819)
Supplement: Supplementary file 1 [file molecules-29-03819-s001.zip › molecules-3100127-supplementary.pdf]

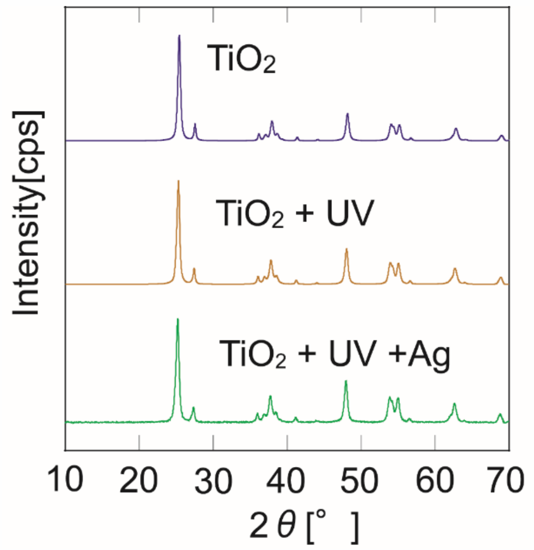

Figure S1. XRD patterns of TiO_2 (as received), the one after addition of Ag NPs and/or UV irradiation.

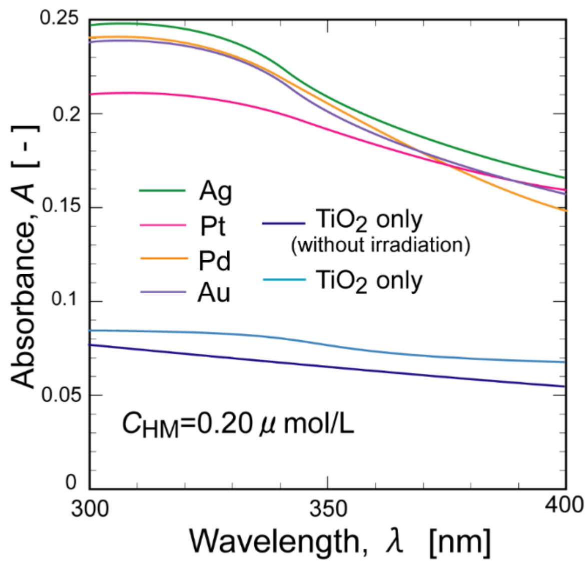

Figure S2. UV-vis spectra of TiO_2 suspension containing heavy metal NPs after US and UV irradiation.

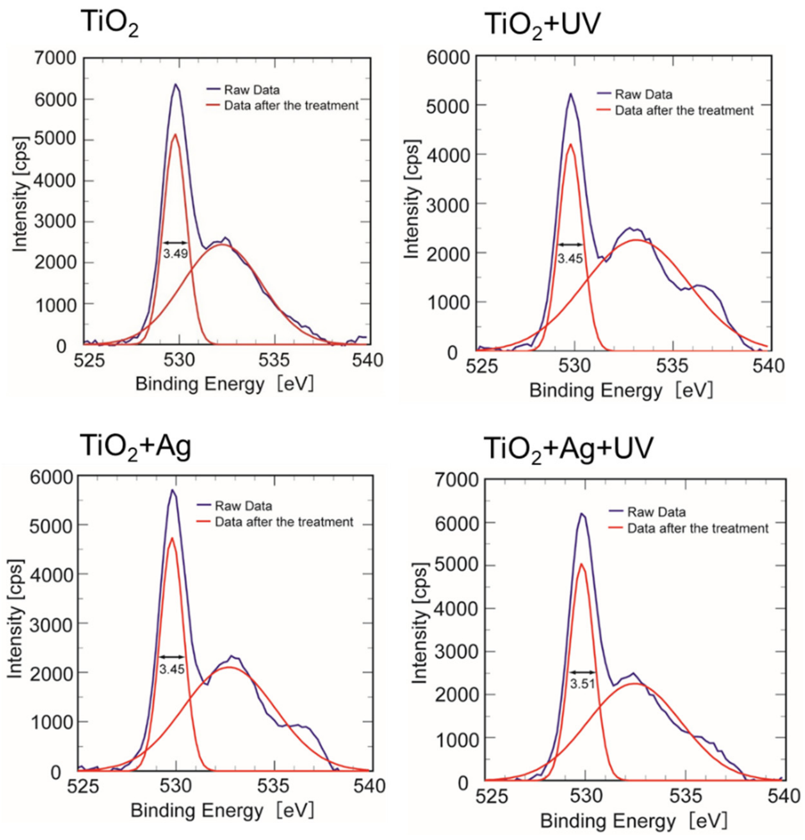

Figure S3. XPS spectra of O 1s of a series of TiO_2 sample.

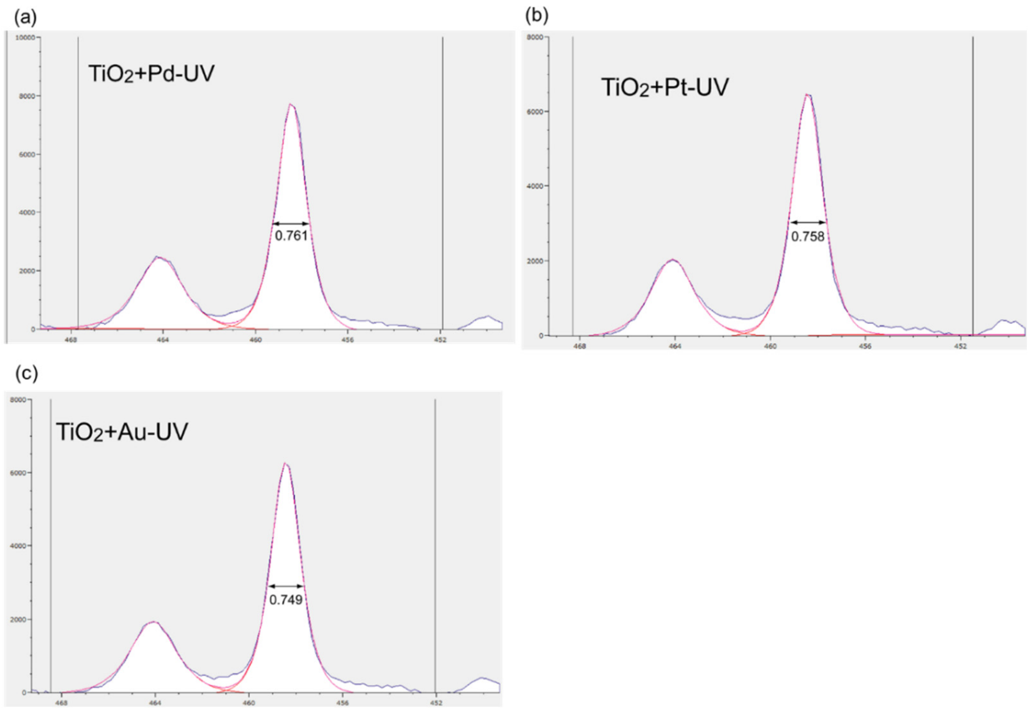

Figure S4. XPS spectrum of Ti 2p peak for samples of TiO_2 suspension containing Pd, Pt, or Au NPs after UV irradiation.

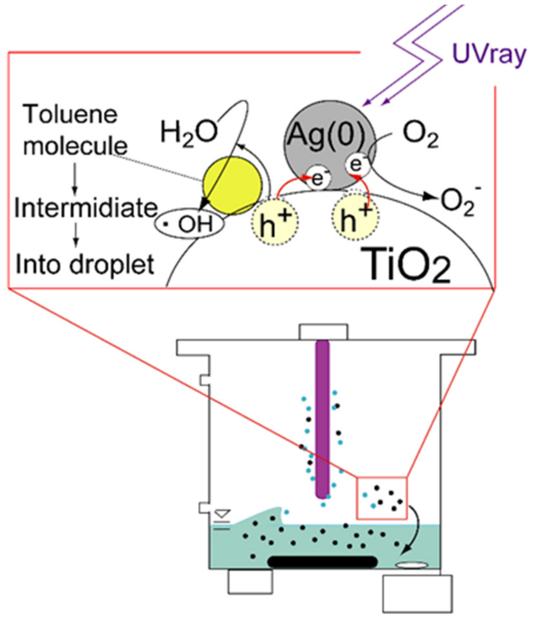

Figure S5. A possible role of Ag NPs in enhancement of toluene degradation reaction in US-generated suspension containing TiO_2 under UV irradiation.

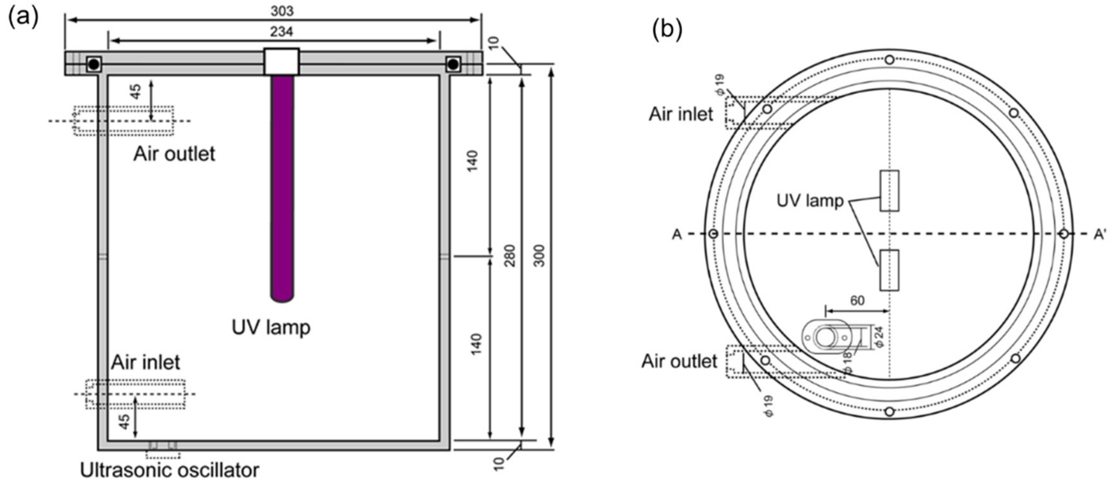

Figure S6. Dimensional diagram of the photocatalytic reactor. (a) Vertical cross-sectional view (A-A' section). (b) Top view.

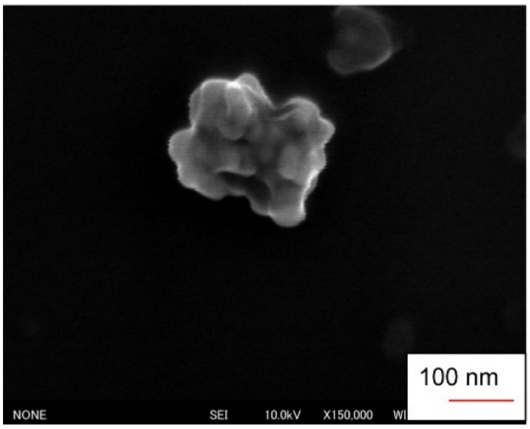

Figure S7. SEM image of TiO_2 (as-received).

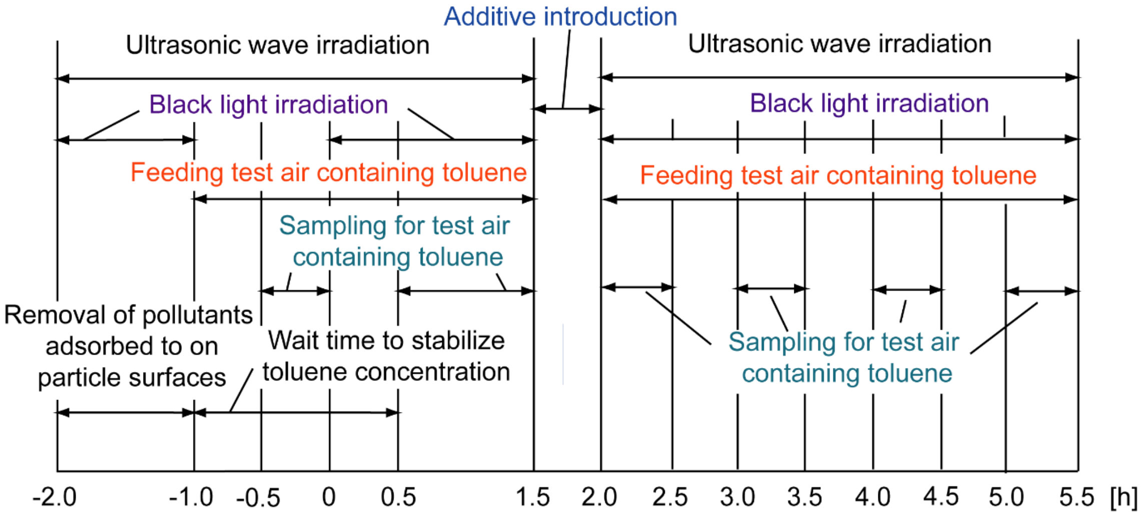

Figure S8. Time schedule of each run of the photocatalytic degradation experiments with feeding of additives into TiO_2 suspension.

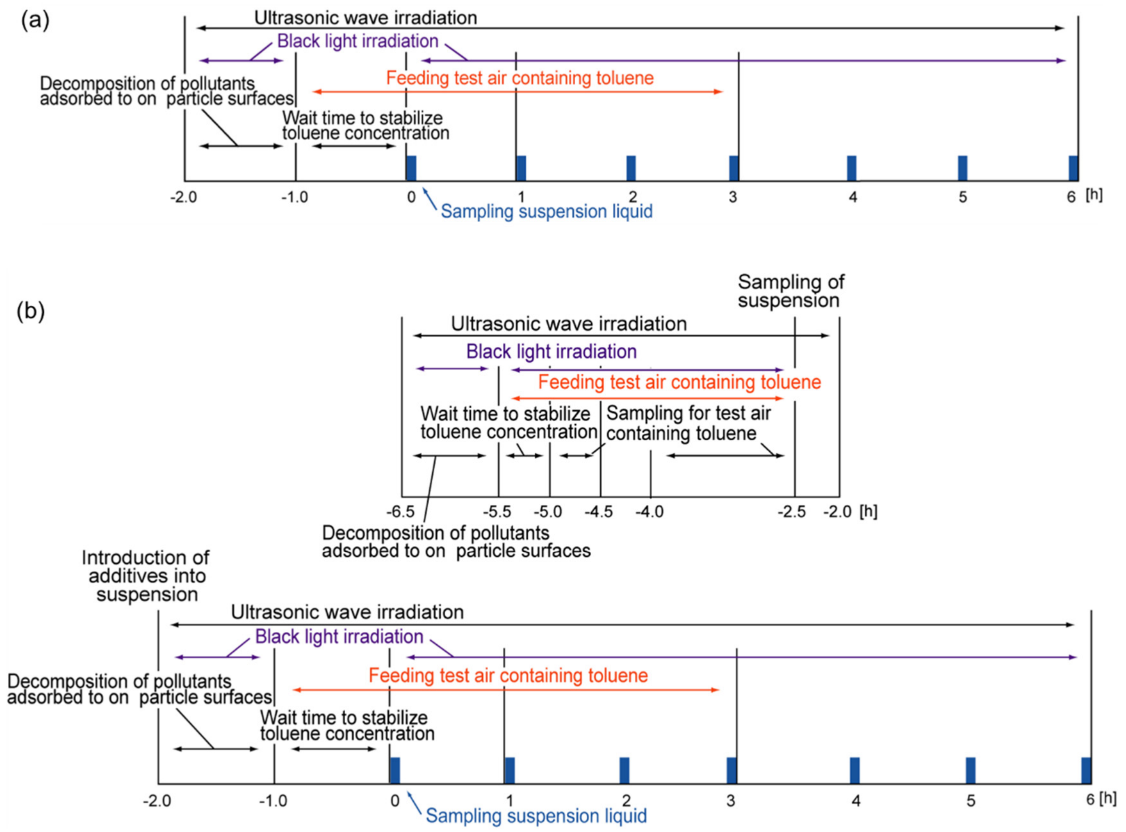

Figure S9. Time schedule of each run of experiments on sampling for WSOC concentration determination (a) without or (b) with additive.

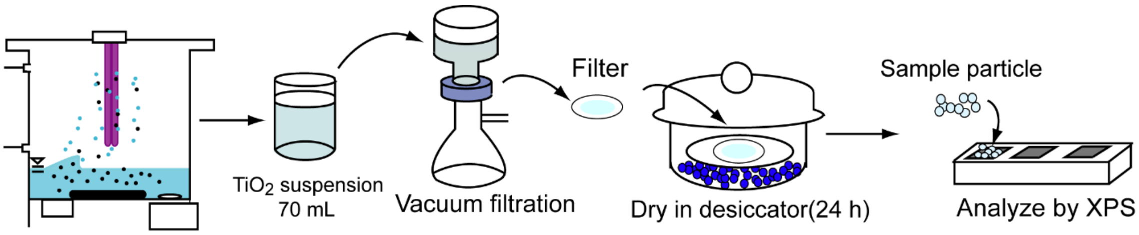

Figure S10. Procedures to prepare samples for analyses by XPS analyzer.
